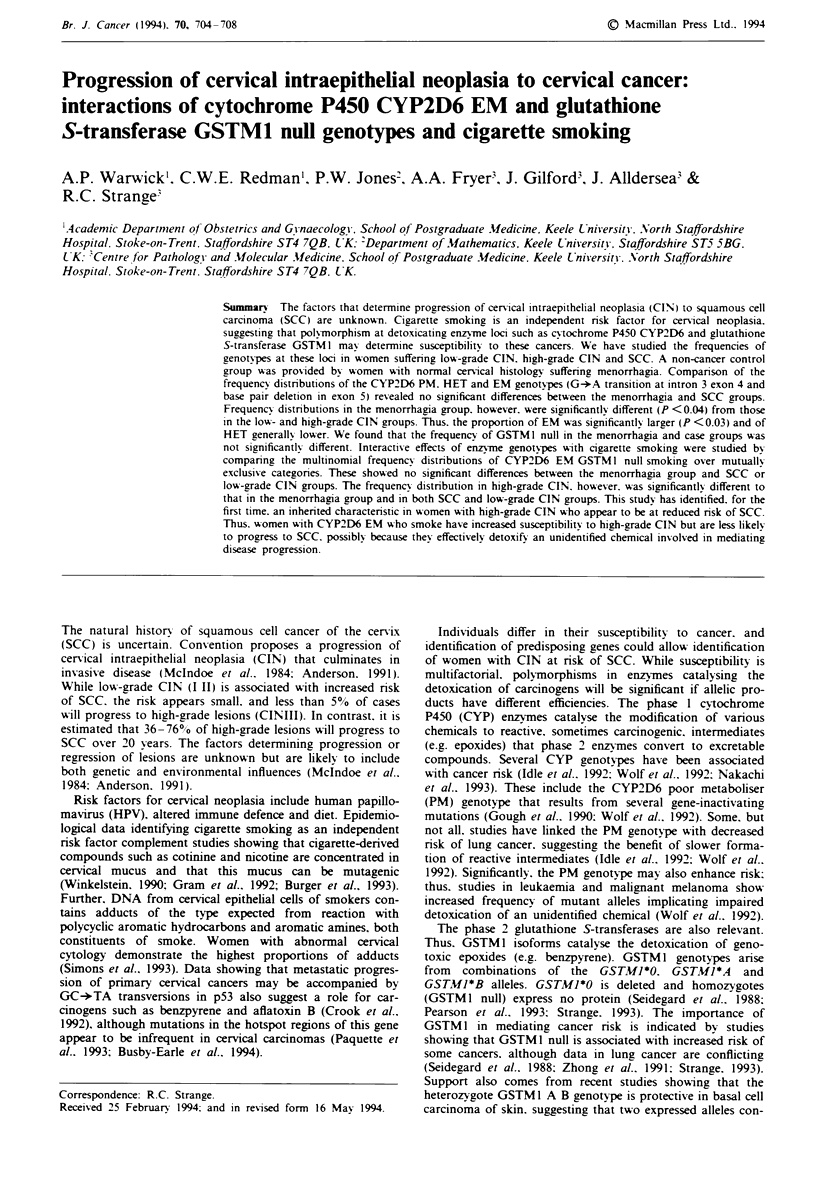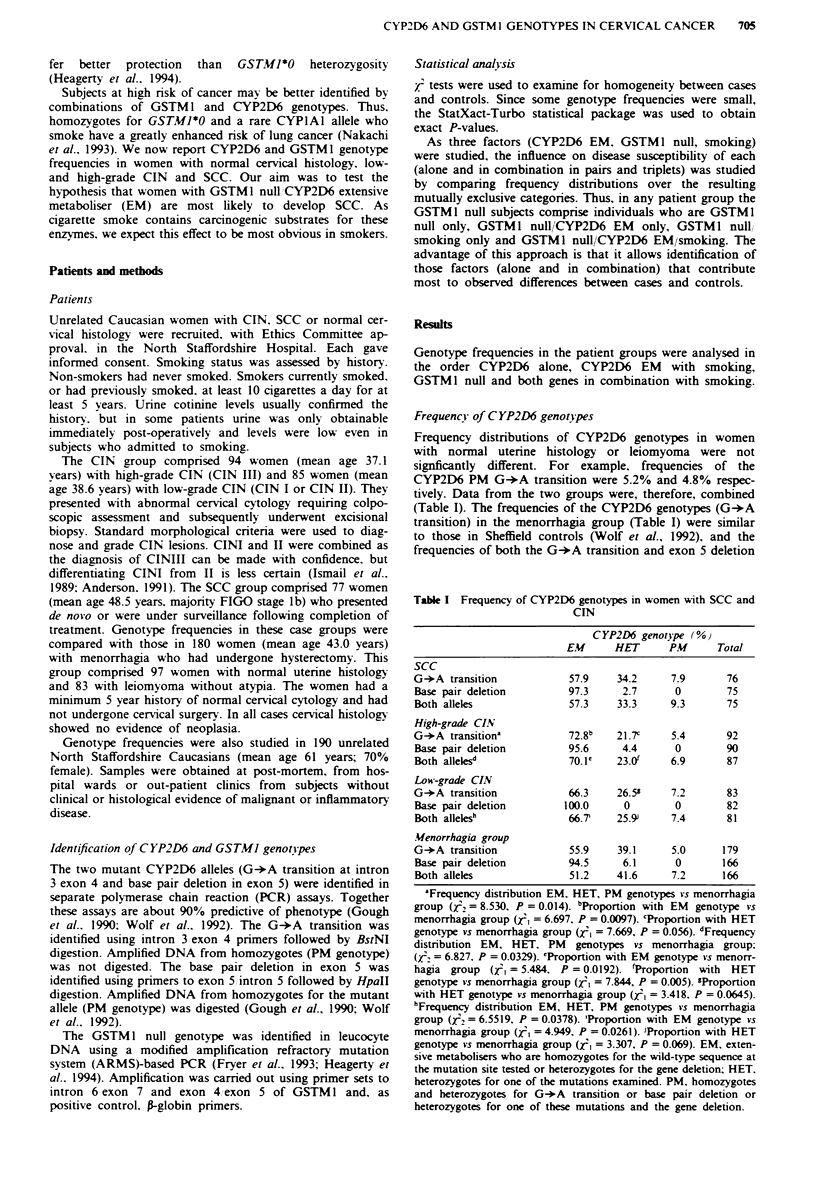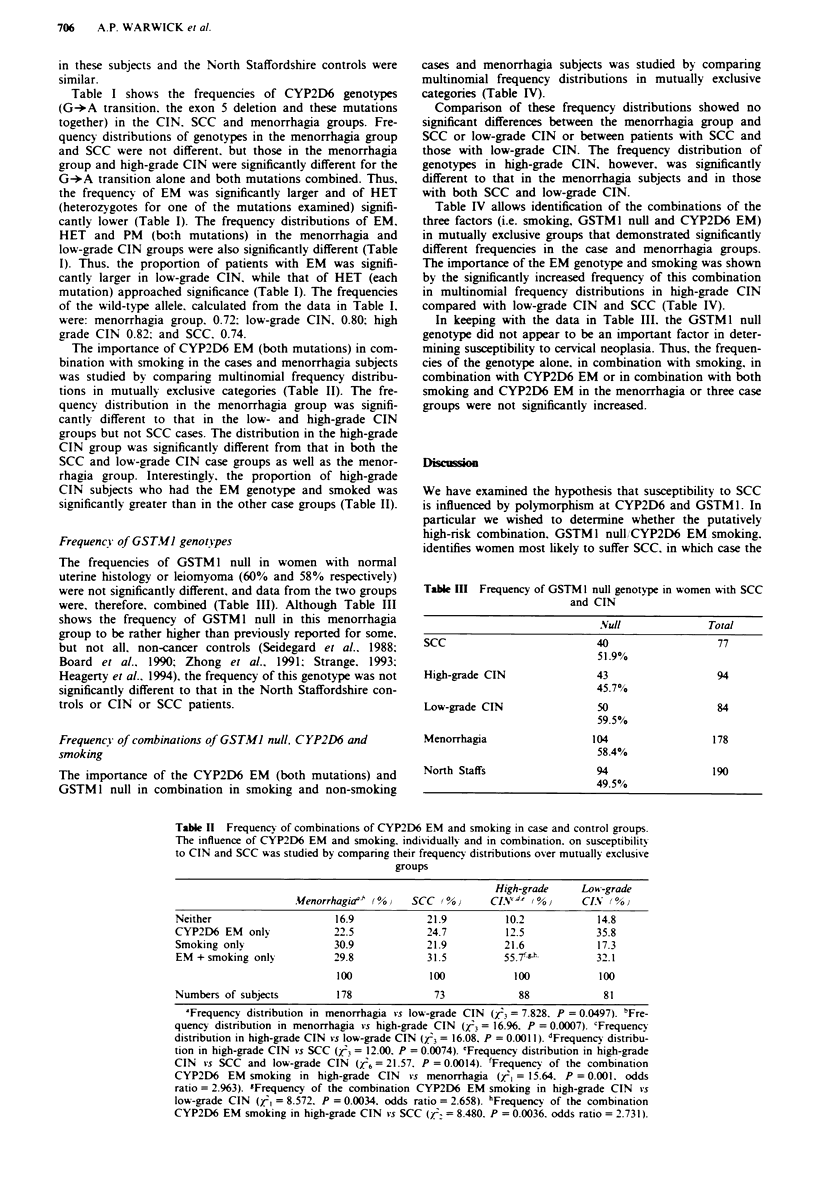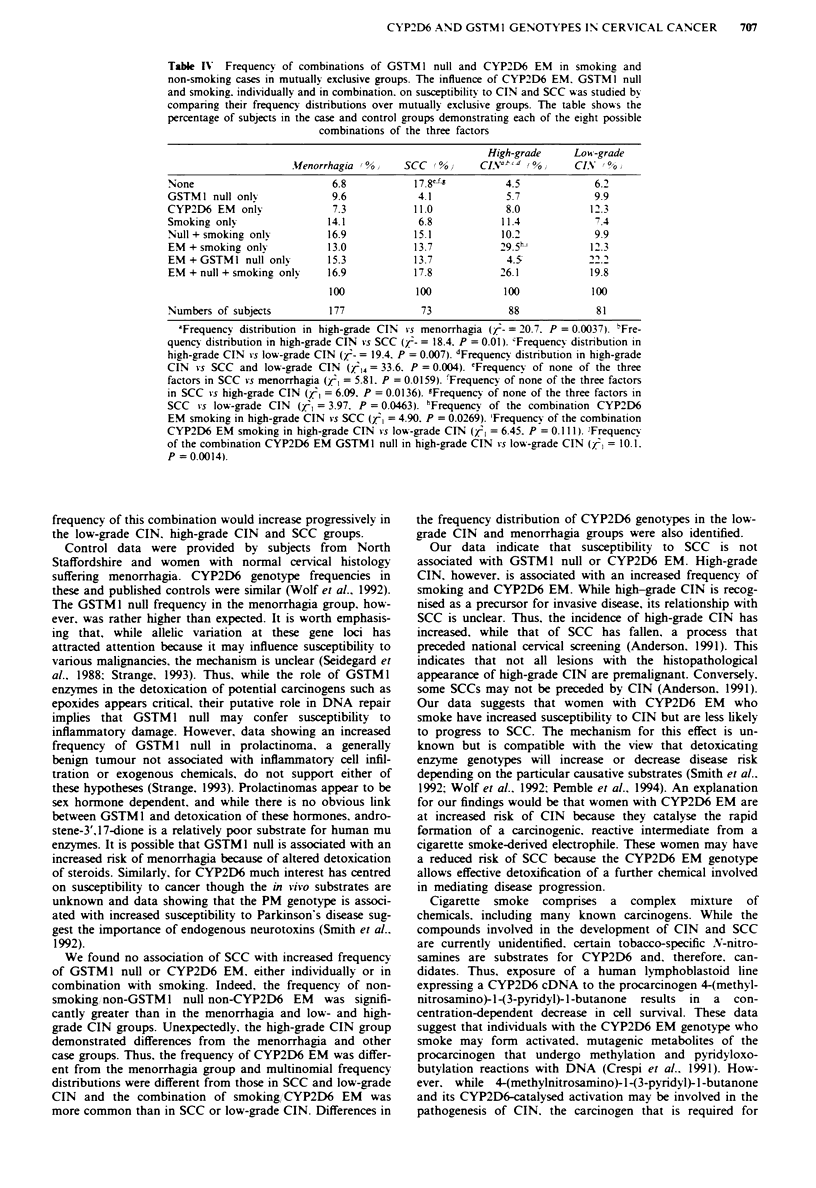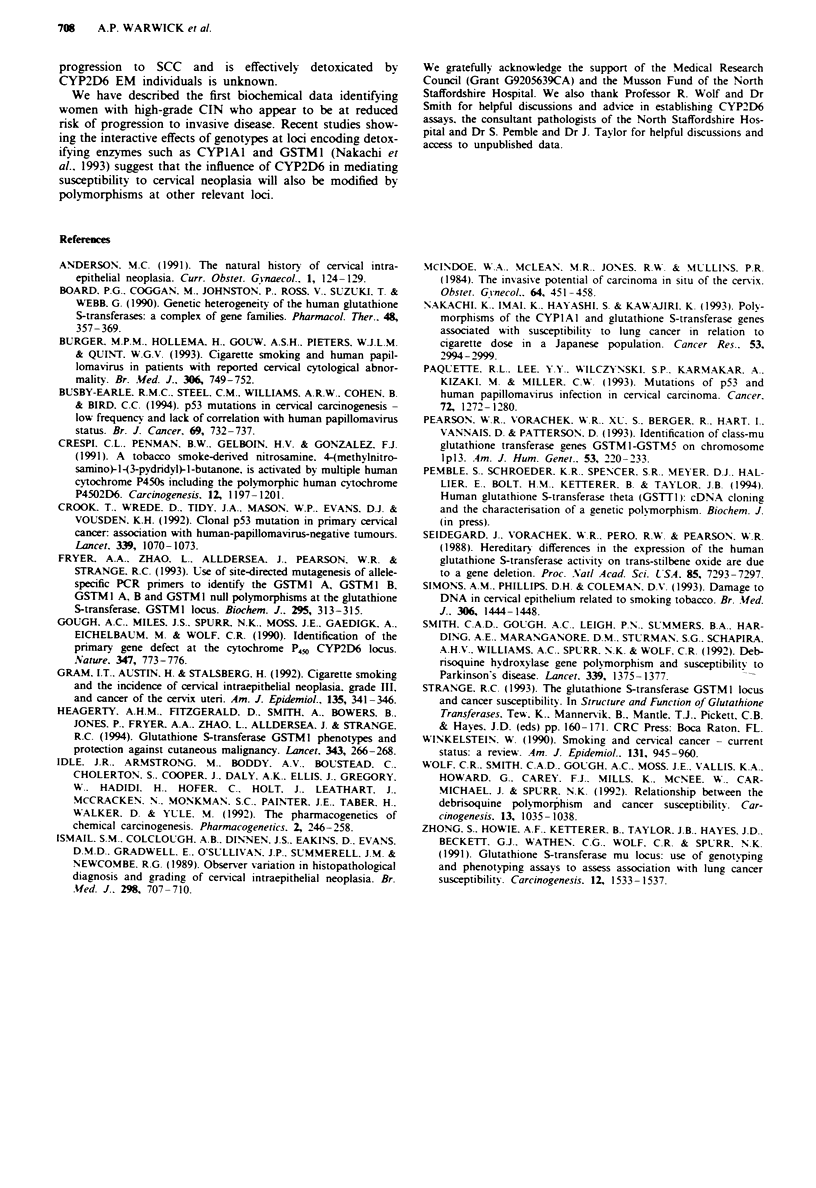# Progression of cervical intraepithelial neoplasia to cervical cancer: interactions of cytochrome P450 CYP2D6 EM and glutathione s-transferase GSTM1 null genotypes and cigarette smoking.

**DOI:** 10.1038/bjc.1994.378

**Published:** 1994-10

**Authors:** A. P. Warwick, C. W. Redman, P. W. Jones, A. A. Fryer, J. Gilford, J. Alldersea, R. C. Strange

**Affiliations:** Academic Department of Obstetrics and Gynaecology, School of Postgraduate Medicine, Keele University, North Staffordshire Hospital, Stoke-on-Trent, UK.

## Abstract

The factors that determine progression of cervical intraepithelial neoplasia (CIN) to squamous cell carcinoma (SCC) are unknown. Cigarette smoking is an independent risk factor for cervical neoplasia, suggesting that polymorphism at detoxicating enzyme loci such as cytochrome P450 CYP2D6 and glutathione S-transferase GSTM1 may determine susceptibility to these cancers. We have studied the frequencies of genotypes at these loci in women suffering low-grade CIN, high-grade CIN and SCC. A non-cancer control group was provided by women with normal cervical histology suffering menorrhagia. Comparison of the frequency distributions of the CYP2D6 PM, HET and EM genotypes (G-->A transition at intron 3/exon 4 and base pair deletion in exon 5) revealed no significant differences between the menorrhagia and SCC groups. Frequency distributions in the menorrhagia group, however, were significantly different (P < 0.04) from those in the low- and high-grade CIN groups. Thus, the proportion of EM was significantly larger (P < 0.03) and of HET generally lower. We found that the frequency of GSTM1 null in the menorrhagia and case groups was not significantly different. Interactive effects of enzyme genotypes with cigarette smoking were studied by comparing the multinomial frequency distributions of CYP2D6 EM/GSTM1 null/smoking over mutually exclusive categories. These showed no significant differences between the menorrhagia group and SCC or low-grade CIN groups. The frequency distribution in high-grade CIN, however, was significantly different to that in the menorrhagia group and in both SCC and low-grade CIN groups. This study was identified, for the first time, an inherited characteristic in women with high-grade CIN who appear to be at reduced risk of SCC. Thus, women with CYP2D6 EM who smoke have increased susceptibility to high-grade CIN but are less likely to progress to SCC, possibly because they effectively detoxify an unidentified chemical involved in mediating disease progression.


Br. J. Cancer (1994). 70, 704-708 © Macmillan Press Ltd., 1994

### Progression of cervical intraepithelial neoplasia to cervical cancer: interactions of cytochrome P450 CYP2D6 EM and glutathione S-transferase GSTM1 null genotypes and cigarette smoking

A.P. Warwick[1], C.W.E. Redman[1], P.W. Jones[2], A.A. Fryer[3], J. Gilford[3], J. Alldersea[3] & R.C. Strange[3]

[1]Academic Department of Obstetrics and Gynaecology, School of Postgraduate Medicine, Keele University, North Staffordshire Hospital, Stoke-on-Trent, Staffordshire ST4 7QB, UK; [2]Department of Mathematics, Keele University, Staffordshire ST5 5BG, UK; [3]Centre for Pathology and Molecular Medicine, School of Postgraduate Medicine, Keele University, North Staffordshire Hospital, Stoke-on-Trent, Staffordshire ST4 7QB, UK.

**Summary** The factors that determine progression of cervical intraepithelial neoplasia (CIN) to squamous cell carcinoma (SCC) are unknown. Cigarette smoking is an independent risk factor for cervical neoplasia, suggesting that polymorphism at detoxicating enzyme loci such as cytochrome P450 CYP2D6 and glutathione S-transferase GSTM1 may determine susceptibility to these cancers. We have studied the frequencies of genotypes at these loci in women suffering low-grade CIN, high-grade CIN and SCC. A non-cancer control group was provided by women with normal cervical histology suffering menorrhagia. Comparison of the frequency distributions of the CYP2D6 PM, HET and EM genotypes (G→A transition at intron 3 exon 4 and base pair deletion in exon 5) revealed no significant differences between the menorrhagia and SCC groups. Frequency distributions in the menorrhagia group, however, were significantly different ($P < 0.04$) from those in the low- and high-grade CIN groups. Thus, the proportion of EM was significantly larger ($P < 0.03$) and of HET generally lower. We found that the frequency of GSTM1 null in the menorrhagia and case groups was not significantly different. Interactive effects of enzyme genotypes with cigarette smoking were studied by comparing the multinomial frequency distributions of CYP2D6 EM GSTM1 null smoking over mutually exclusive categories. These showed no significant differences between the menorrhagia group and SCC or low-grade CIN groups. The frequency distribution in high-grade CIN, however, was significantly different to that in the menorrhagia group and in both SCC and low-grade CIN groups. This study has identified, for the first time, an inherited characteristic in women with high-grade CIN who appear to be at reduced risk of SCC. Thus, women with CYP2D6 EM who smoke have increased susceptibility to high-grade CIN but are less likely to progress to SCC, possibly because they effectively detoxify an unidentified chemical involved in mediating disease progression.

The natural history of squamous cell cancer of the cervix (SCC) is uncertain. Convention proposes a progression of cervical intraepithelial neoplasia (CIN) that culminates in invasive disease (McIndoe et al., 1984; Anderson, 1991). While low-grade CIN (I II) is associated with increased risk of SCC, the risk appears small, and less than 5% of cases will progress to high-grade lesions (CINIII). In contrast, it is estimated that 36-76% of high-grade lesions will progress to SCC over 20 years. The factors determining progression or regression of lesions are unknown but are likely to include both genetic and environmental influences (McIndoe et al., 1984; Anderson, 1991).

Risk factors for cervical neoplasia include human papillomavirus (HPV), altered immune defence and diet. Epidemiological data identifying cigarette smoking as an independent risk factor complement studies showing that cigarette-derived compounds such as cotinine and nicotine are concentrated in cervical mucus and that this mucus can be mutagenic (Winkelstein, 1990; Gram et al., 1992; Burger et al., 1993). Further, DNA from cervical epithelial cells of smokers contains adducts of the type expected from reaction with polycyclic aromatic hydrocarbons and aromatic amines, both constituents of smoke. Women with abnormal cervical cytology demonstrate the highest proportions of adducts (Simons et al., 1993). Data showing that metastatic progression of primary cervical cancers may be accompanied by GC→TA transversions in p53 also suggest a role for carcinogens such as benzpyrene and aflatoxin B (Crook et al., 1992), although mutations in the hotspot regions of this gene appear to be infrequent in cervical carcinomas (Paquette et al., 1993; Busby-Earle et al., 1994).

Correspondence: R.C. Strange.
Received 25 February 1994; and in revised form 16 May 1994.

Individuals differ in their susceptibility to cancer, and identification of predisposing genes could allow identification of women with CIN at risk of SCC. While susceptibility is multifactorial, polymorphisms in enzymes catalysing the detoxication of carcinogens will be significant if allelic products have different efficiencies. The phase 1 cytochrome P450 (CYP) enzymes catalyse the modification of various chemicals to reactive, sometimes carcinogenic, intermediates (e.g. epoxides) that phase 2 enzymes convert to excretable compounds. Several CYP genotypes have been associated with cancer risk (Idle et al., 1992; Wolf et al., 1992; Nakachi et al., 1993). These include the CYP2D6 poor metaboliser (PM) genotype that results from several gene-inactivating mutations (Gough et al., 1990; Wolf et al., 1992). Some, but not all, studies have linked the PM genotype with decreased risk of lung cancer, suggesting the benefit of slower formation of reactive intermediates (Idle et al., 1992; Wolf et al., 1992). Significantly, the PM genotype may also enhance risk; thus, studies in leukaemia and malignant melanoma show increased frequency of mutant alleles implicating impaired detoxication of an unidentified chemical (Wolf et al., 1992).

The phase 2 glutathione S-transferases are also relevant. Thus, GSTM1 isoforms catalyse the detoxication of genotoxic epoxides (e.g. benzpyrene). GSTM1 genotypes arise from combinations of the *GSTM1*0*, *GSTM1*A* and *GSTM1*B* alleles. *GSTM1*0* is deleted and homozygotes (GSTM1 null) express no protein (Seidegard et al., 1988; Pearson et al., 1993; Strange, 1993). The importance of GSTM1 in mediating cancer risk is indicated by studies showing that GSTM1 null is associated with increased risk of some cancers, although data in lung cancer are conflicting (Seidegard et al., 1988; Zhong et al., 1991; Strange, 1993). Support also comes from recent studies showing that the heterozygote GSTM1 A B genotype is protective in basal cell carcinoma of skin, suggesting that two expressed alleles confer better protection than *GSTM1*0* heterozygosity (Heagerty *et al.*, 1994).

Subjects at high risk of cancer may be better identified by combinations of GSTM1 and CYP2D6 genotypes. Thus, homozygotes for *GSTM1*0* and a rare CYP1A1 allele who smoke have a greatly enhanced risk of lung cancer (Nakachi *et al.*, 1993). We now report CYP2D6 and GSTM1 genotype frequencies in women with normal cervical histology, low- and high-grade CIN and SCC. Our aim was to test the hypothesis that women with GSTM1 null/CYP2D6 extensive metaboliser (EM) are most likely to develop SCC. As cigarette smoke contains carcinogenic substrates for these enzymes, we expect this effect to be most obvious in smokers.

### Patients and methods

### *Patients*

Unrelated Caucasian women with CIN, SCC or normal cervical histology were recruited, with Ethics Committee approval, in the North Staffordshire Hospital. Each gave informed consent. Smoking status was assessed by history. Non-smokers had never smoked. Smokers currently smoked, or had previously smoked, at least 10 cigarettes a day for at least 5 years. Urine cotinine levels usually confirmed the history, but in some patients urine was only obtainable immediately post-operatively and levels were low even in subjects who admitted to smoking.

The CIN group comprised 94 women (mean age 37.1 years) with high-grade CIN (CIN III) and 85 women (mean age 38.6 years) with low-grade CIN (CIN I or CIN II). They presented with abnormal cervical cytology requiring colposcopic assessment and subsequently underwent excisional biopsy. Standard morphological criteria were used to diagnose and grade CIN lesions. CINI and II were combined as the diagnosis of CINIII can be made with confidence, but differentiating CINI from II is less certain (Ismail *et al.*, 1989; Anderson, 1991). The SCC group comprised 77 women (mean age 48.5 years, majority FIGO stage 1b) who presented *de novo* or were under surveillance following completion of treatment. Genotype frequencies in these case groups were compared with those in 180 women (mean age 43.0 years) with menorrhagia who had undergone hysterectomy. This group comprised 97 women with normal uterine histology and 83 with leiomyoma without atypia. The women had a minimum 5 year history of normal cervical cytology and had not undergone cervical surgery. In all cases cervical histology showed no evidence of neoplasia.

Genotype frequencies were also studied in 190 unrelated North Staffordshire Caucasians (mean age 61 years; 70% female). Samples were obtained at post-mortem, from hospital wards or out-patient clinics from subjects without clinical or histological evidence of malignant or inflammatory disease.

### *Identification of CYP2D6 and GSTM1 genotypes*

The two mutant CYP2D6 alleles (G→A transition at intron 3/exon 4 and base pair deletion in exon 5) were identified in separate polymerase chain reaction (PCR) assays. Together these assays are about 90% predictive of phenotype (Gough *et al.*, 1990; Wolf *et al.*, 1992). The G→A transition was identified using intron 3/exon 4 primers followed by *Bst*NI digestion. Amplified DNA from homozygotes (PM genotype) was not digested. The base pair deletion in exon 5 was identified using primers to exon 5/intron 5 followed by *Hpa*II digestion. Amplified DNA from homozygotes for the mutant allele (PM genotype) was digested (Gough *et al.*, 1990; Wolf *et al.*, 1992).

The GSTM1 null genotype was identified in leucocyte DNA using a modified amplification refractory mutation system (ARMS)-based PCR (Fryer *et al.*, 1993; Heagerty *et al.*, 1994). Amplification was carried out using primer sets to intron 6/exon 7 and exon 4/exon 5 of GSTM1 and, as positive control, β-globin primers.

### *Statistical analysis*

$\chi^2$ tests were used to examine for homogeneity between cases and controls. Since some genotype frequencies were small, the StatXact-Turbo statistical package was used to obtain exact *P*-values.

As three factors (CYP2D6 EM, GSTM1 null, smoking) were studied, the influence on disease susceptibility of each (alone and in combination in pairs and triplets) was studied by comparing frequency distributions over the resulting mutually exclusive categories. Thus, in any patient group the GSTM1 null subjects comprise individuals who are GSTM1 null only, GSTM1 null/CYP2D6 EM only, GSTM1 null/smoking only and GSTM1 null/CYP2D6 EM/smoking. The advantage of this approach is that it allows identification of those factors (alone and in combination) that contribute most to observed differences between cases and controls.

### Results

Genotype frequencies in the patient groups were analysed in the order CYP2D6 alone, CYP2D6 EM with smoking, GSTM1 null and both genes in combination with smoking.

### *Frequency of CYP2D6 genotypes*

Frequency distributions of CYP2D6 genotypes in women with normal uterine histology or leiomyoma were not signficantly different. For example, frequencies of the CYP2D6 PM G→A transition were 5.2% and 4.8% respectively. Data from the two groups were, therefore, combined (Table I). The frequencies of the CYP2D6 genotypes (G→A transition) in the menorrhagia group (Table I) were similar to those in Sheffield controls (Wolf *et al.*, 1992), and the frequencies of both the G→A transition and exon 5 deletion

**Table I** Frequency of CYP2D6 genotypes in women with SCC and CIN

| | *CYP2D6 genotype (%)* | | | |
|---|---|---|---|---|
| | *EM* | *HET* | *PM* | *Total* |
| *SCC* | | | | |
| G→A transition | 57.9 | 34.2 | 7.9 | 76 |
| Base pair deletion | 97.3 | 2.7 | 0 | 75 |
| Both alleles | 57.3 | 33.3 | 9.3 | 75 |
| *High-grade CIN* | | | | |
| G→A transition[a] | 72.8[b] | 21.7[c] | 5.4 | 92 |
| Base pair deletion | 95.6 | 4.4 | 0 | 90 |
| Both alleles[d] | 70.1[e] | 23.0[f] | 6.9 | 87 |
| *Low-grade CIN* | | | | |
| G→A transition | 66.3 | 26.5[g] | 7.2 | 83 |
| Base pair deletion | 100.0 | 0 | 0 | 82 |
| Both alleles[h] | 66.7[i] | 25.9[j] | 7.4 | 81 |
| *Menorrhagia group* | | | | |
| G→A transition | 55.9 | 39.1 | 5.0 | 179 |
| Base pair deletion | 94.5 | 6.1 | 0 | 166 |
| Both alleles | 51.2 | 41.6 | 7.2 | 166 |

[a]Frequency distribution EM, HET, PM genotypes *vs* menorrhagia group ($\chi^2_2 = 8.530$, $P = 0.014$). [b]Proportion with EM genotype *vs* menorrhagia group ($\chi^2_1 = 6.697$, $P = 0.0097$). [c]Proportion with HET genotype *vs* menorrhagia group ($\chi^2_1 = 7.669$, $P = 0.056$). [d]Frequency distribution EM, HET, PM genotypes *vs* menorrhagia group; ($\chi^2_2 = 6.827$, $P = 0.0329$). [e]Proportion with EM genotype *vs* menorrhagia group ($\chi^2_1 = 5.484$, $P = 0.0192$). [f]Proportion with HET genotype *vs* menorrhagia group ($\chi^2_1 = 7.844$, $P = 0.005$). [g]Proportion with HET genotype *vs* menorrhagia group ($\chi^2_1 = 3.418$, $P = 0.0645$). [h]Frequency distribution EM, HET, PM genotypes *vs* menorrhagia group ($\chi^2_2 = 6.5519$, $P = 0.0378$). [i]Proportion with EM genotype *vs* menorrhagia group ($\chi^2_1 = 4.949$, $P = 0.0261$). [j]Proportion with HET genotype *vs* menorrhagia group ($\chi^2_1 = 3.307$, $P = 0.069$). EM, extensive metabolisers who are homozygotes for the wild-type sequence at the mutation site tested or heterozygotes for the gene deletion; HET, heterozygotes for one of the mutations examined. PM, homozygotes and heterozygotes for G→A transition or base pair deletion or heterozygotes for one of these mutations and the gene deletion.

in these subjects and the North Staffordshire controls were similar.

Table I shows the frequencies of CYP2D6 genotypes (G→A transition, the exon 5 deletion and these mutations together) in the CIN, SCC and menorrhagia groups. Frequency distributions of genotypes in the menorrhagia group and SCC were not different, but those in the menorrhagia group and high-grade CIN were significantly different for the G→A transition alone and both mutations combined. Thus, the frequency of EM was significantly larger and of HET (heterozygotes for one of the mutations examined) significantly lower (Table I). The frequency distributions of EM, HET and PM (both mutations) in the menorrhagia and low-grade CIN groups were also significantly different (Table I). Thus, the proportion of patients with EM was significantly larger in low-grade CIN, while that of HET (each mutation) approached significance (Table I). The frequencies of the wild-type allele, calculated from the data in Table I, were: menorrhagia group, 0.72; low-grade CIN, 0.80; high grade CIN 0.82; and SCC, 0.74.

The importance of CYP2D6 EM (both mutations) in combination with smoking in the cases and menorrhagia subjects was studied by comparing multinomial frequency distributions in mutually exclusive categories (Table II). The frequency distribution in the menorrhagia group was significantly different to that in the low- and high-grade CIN groups but not SCC cases. The distribution in the high-grade CIN group was significantly different from that in both the SCC and low-grade CIN case groups as well as the menorrhagia group. Interestingly, the proportion of high-grade CIN subjects who had the EM genotype and smoked was significantly greater than in the other case groups (Table II).

### *Frequency of GSTM1 genotypes*

The frequencies of GSTM1 null in women with normal uterine histology or leiomyoma (60% and 58% respectively) were not significantly different, and data from the two groups were, therefore, combined (Table III). Although Table III shows the frequency of GSTM1 null in this menorrhagia group to be rather higher than previously reported for some, but not all, non-cancer controls (Seidegard *et al.*, 1988; Board *et al.*, 1990; Zhong *et al.*, 1991; Strange, 1993; Heagerty *et al.*, 1994), the frequency of this genotype was not significantly different to that in the North Staffordshire controls or CIN or SCC patients.

### *Frequency of combinations of GSTM1 null, CYP2D6 and smoking*

The importance of the CYP2D6 EM (both mutations) and GSTM1 null in combination in smoking and non-smoking cases and menorrhagia subjects was studied by comparing multinomial frequency distributions in mutually exclusive categories (Table IV).

Comparison of these frequency distributions showed no significant differences between the menorrhagia group and SCC or low-grade CIN or between patients with SCC and those with low-grade CIN. The frequency distribution of genotypes in high-grade CIN, however, was significantly different to that in the menorrhagia subjects and in those with both SCC and low-grade CIN.

Table IV allows identification of the combinations of the three factors (i.e. smoking, GSTM1 null and CYP2D6 EM) in mutually exclusive groups that demonstrated significantly different frequencies in the case and menorrhagia groups. The importance of the EM genotype and smoking was shown by the significantly increased frequency of this combination in multinomial frequency distributions in high-grade CIN compared with low-grade CIN and SCC (Table IV).

In keeping with the data in Table III, the GSTM1 null genotype did not appear to be an important factor in determining susceptibility to cervical neoplasia. Thus, the frequencies of the genotype alone, in combination with smoking, in combination with CYP2D6 EM or in combination with both smoking and CYP2D6 EM in the menorrhagia or three case groups were not significantly increased.

### Discussion

We have examined the hypothesis that susceptibility to SCC is influenced by polymorphism at CYP2D6 and GSTM1. In particular we wished to determine whether the putatively high-risk combination, GSTM1 null/CYP2D6 EM/smoking, identifies women most likely to suffer SCC, in which case the

**Table III** Frequency of GSTM1 null genotype in women with SCC and CIN

| | *Null* | *Total* |
|---|---|---|
| SCC | 40<br>51.9% | 77 |
| High-grade CIN | 43<br>45.7% | 94 |
| Low-grade CIN | 50<br>59.5% | 84 |
| Menorrhagia | 104<br>58.4% | 178 |
| North Staffs | 94<br>49.5% | 190 |

**Table II** Frequency of combinations of CYP2D6 EM and smoking in case and control groups. The influence of CYP2D6 EM and smoking, individually and in combination, on susceptibility to CIN and SCC was studied by comparing their frequency distributions over mutually exclusive groups

| | *Menorrhagia*[a,b] (%) | *SCC* (%) | *High-grade CIN*[c,d,e] (%) | *Low-grade CIN* (%) |
|---|---|---|---|---|
| Neither | 16.9 | 21.9 | 10.2 | 14.8 |
| CYP2D6 EM only | 22.5 | 24.7 | 12.5 | 35.8 |
| Smoking only | 30.9 | 21.9 | 21.6 | 17.3 |
| EM + smoking only | 29.8 | 31.5 | 55.7[f,g,h] | 32.1 |
| | 100 | 100 | 100 | 100 |
| Numbers of subjects | 178 | 73 | 88 | 81 |

[a]Frequency distribution in menorrhagia *vs* low-grade CIN ($\chi^2_3 = 7.828$, $P = 0.0497$). [b]Frequency distribution in menorrhagia *vs* high-grade CIN ($\chi^2_3 = 16.96$, $P = 0.0007$). [c]Frequency distribution in high-grade CIN *vs* low-grade CIN ($\chi^2_3 = 16.08$, $P = 0.0011$). [d]Frequency distribution in high-grade CIN *vs* SCC ($\chi^2_3 = 12.00$, $P = 0.0074$). [e]Frequency distribution in high-grade CIN *vs* SCC and low-grade CIN ($\chi^2_6 = 21.57$, $P = 0.0014$). [f]Frequency of the combination CYP2D6 EM smoking in high-grade CIN *vs* menorrhagia ($\chi^2_1 = 15.64$, $P = 0.001$, odds ratio = 2.963). [g]Frequency of the combination CYP2D6 EM smoking in high-grade CIN *vs* low-grade CIN ($\chi^2_1 = 8.572$, $P = 0.0034$, odds ratio = 2.658). [h]Frequency of the combination CYP2D6 EM smoking in high-grade CIN *vs* SCC ($\chi^2_2 = 8.480$, $P = 0.0036$, odds ratio = 2.731).

**Table IV** Frequency of combinations of GSTM1 null and CYP2D6 EM in smoking and non-smoking cases in mutually exclusive groups. The influence of CYP2D6 EM, GSTM1 null and smoking, individually and in combination, on susceptibility to CIN and SCC was studied by comparing their frequency distributions over mutually exclusive groups. The table shows the percentage of subjects in the case and control groups demonstrating each of the eight possible combinations of the three factors

| | *Menorrhagia (%)* | *SCC (%)* | *High-grade CIN*[a,b,c,d] *(%)* | *Low-grade CIN (%)* |
|---|---|---|---|---|
| None | 6.8 | 17.8[e,f,g] | 4.5 | 6.2 |
| GSTM1 null only | 9.6 | 4.1 | 5.7 | 9.9 |
| CYP2D6 EM only | 7.3 | 11.0 | 8.0 | 12.3 |
| Smoking only | 14.1 | 6.8 | 11.4 | 7.4 |
| Null + smoking only | 16.9 | 15.1 | 10.2 | 9.9 |
| EM + smoking only | 13.0 | 13.7 | 29.5[h,i] | 12.3 |
| EM + GSTM1 null only | 15.3 | 13.7 | 4.5[j] | 22.2 |
| EM + null + smoking only | 16.9 | 17.8 | 26.1 | 19.8 |
| | 100 | 100 | 100 | 100 |
| Numbers of subjects | 177 | 73 | 88 | 81 |

[a]Frequency distribution in high-grade CIN *vs* menorrhagia ($\chi^2 = 20.7$, $P = 0.0037$). [b]Frequency distribution in high-grade CIN *vs* SCC ($\chi^2 = 18.4$, $P = 0.01$). [c]Frequency distribution in high-grade CIN *vs* low-grade CIN ($\chi^2 = 19.4$, $P = 0.007$). [d]Frequency distribution in high-grade CIN *vs* SCC and low-grade CIN ($\chi^2_{14} = 33.6$, $P = 0.004$). [e]Frequency of none of the three factors in SCC *vs* menorrhagia ($\chi^2_1 = 5.81$, $P = 0.0159$). [f]Frequency of none of the three factors in SCC *vs* high-grade CIN ($\chi^2_1 = 6.09$, $P = 0.0136$). [g]Frequency of none of the three factors in SCC *vs* low-grade CIN ($\chi^2_1 = 3.97$, $P = 0.0463$). [h]Frequency of the combination CYP2D6 EM smoking in high-grade CIN *vs* SCC ($\chi^2_1 = 4.90$, $P = 0.0269$). [i]Frequency of the combination CYP2D6 EM smoking in high-grade CIN *vs* low-grade CIN ($\chi^2_1 = 6.45$, $P = 0.111$). [j]Frequency of the combination CYP2D6 EM GSTM1 null in high-grade CIN *vs* low-grade CIN ($\chi^2_1 = 10.1$, $P = 0.0014$).

frequency of this combination would increase progressively in the low-grade CIN, high-grade CIN and SCC groups.

Control data were provided by subjects from North Staffordshire and women with normal cervical histology suffering menorrhagia. CYP2D6 genotype frequencies in these and published controls were similar (Wolf *et al.*, 1992). The GSTM1 null frequency in the menorrhagia group, however, was rather higher than expected. It is worth emphasising that, while allelic variation at these gene loci has attracted attention because it may influence susceptibility to various malignancies, the mechanism is unclear (Seidegard *et al.*, 1988; Strange, 1993). Thus, while the role of GSTM1 enzymes in the detoxication of potential carcinogens such as epoxides appears critical, their putative role in DNA repair implies that GSTM1 null may confer susceptibility to inflammatory damage. However, data showing an increased frequency of GSTM1 null in prolactinoma, a generally benign tumour not associated with inflammatory cell infiltration or exogenous chemicals, do not support either of these hypotheses (Strange, 1993). Prolactinomas appear to be sex hormone dependent, and while there is no obvious link between GSTM1 and detoxication of these hormones, androstene-3',17-dione is a relatively poor substrate for human mu enzymes. It is possible that GSTM1 null is associated with an increased risk of menorrhagia because of altered detoxication of steroids. Similarly, for CYP2D6 much interest has centred on susceptibility to cancer though the *in vivo* substrates are unknown and data showing that the PM genotype is associated with increased susceptibility to Parkinson's disease suggest the importance of endogenous neurotoxins (Smith *et al.*, 1992).

We found no association of SCC with increased frequency of GSTM1 null or CYP2D6 EM, either individually or in combination with smoking. Indeed, the frequency of non-smoking/non-GSTM1 null non-CYP2D6 EM was significantly greater than in the menorrhagia and low- and high-grade CIN groups. Unexpectedly, the high-grade CIN group demonstrated differences from the menorrhagia and other case groups. Thus, the frequency of CYP2D6 EM was different from the menorrhagia group and multinomial frequency distributions were different from those in SCC and low-grade CIN and the combination of smoking/CYP2D6 EM was more common than in SCC or low-grade CIN. Differences in the frequency distribution of CYP2D6 genotypes in the low-grade CIN and menorrhagia groups were also identified.

Our data indicate that susceptibility to SCC is not associated with GSTM1 null or CYP2D6 EM. High-grade CIN, however, is associated with an increased frequency of smoking and CYP2D6 EM. While high-grade CIN is recognised as a precursor for invasive disease, its relationship with SCC is unclear. Thus, the incidence of high-grade CIN has increased, while that of SCC has fallen, a process that preceded national cervical screening (Anderson, 1991). This indicates that not all lesions with the histopathological appearance of high-grade CIN are premalignant. Conversely, some SCCs may not be preceded by CIN (Anderson, 1991). Our data suggests that women with CYP2D6 EM who smoke have increased susceptibility to CIN but are less likely to progress to SCC. The mechanism for this effect is unknown but is compatible with the view that detoxicating enzyme genotypes will increase or decrease disease risk depending on the particular causative substrates (Smith *et al.*, 1992; Wolf *et al.*, 1992; Pemble *et al.*, 1994). An explanation for our findings would be that women with CYP2D6 EM are at increased risk of CIN because they catalyse the rapid formation of a carcinogenic, reactive intermediate from a cigarette smoke-derived electrophile. These women may have a reduced risk of SCC because the CYP2D6 EM genotype allows effective detoxification of a further chemical involved in mediating disease progression.

Cigarette smoke comprises a complex mixture of chemicals, including many known carcinogens. While the compounds involved in the development of CIN and SCC are currently unidentified, certain tobacco-specific *N*-nitrosamines are substrates for CYP2D6 and, therefore, candidates. Thus, exposure of a human lymphoblastoid line expressing a CYP2D6 cDNA to the procarcinogen 4-(methylnitrosamino)-1-(3-pyridyl)-1-butanone results in a concentration-dependent decrease in cell survival. These data suggest that individuals with the CYP2D6 EM genotype who smoke may form activated, mutagenic metabolites of the procarcinogen that undergo methylation and pyridyloxobutylation reactions with DNA (Crespi *et al.*, 1991). However, while 4-(methylnitrosamino)-1-(3-pyridyl)-1-butanone and its CYP2D6-catalysed activation may be involved in the pathogenesis of CIN, the carcinogen that is required for progression to SCC and is effectively detoxicated by CYP2D6 EM individuals is unknown.

We have described the first biochemical data identifying women with high-grade CIN who appear to be at reduced risk of progression to invasive disease. Recent studies showing the interactive effects of genotypes at loci encoding detoxifying enzymes such as CYP1A1 and GSTM1 (Nakachi et al., 1993) suggest that the influence of CYP2D6 in mediating susceptibility to cervical neoplasia will also be modified by polymorphisms at other relevant loci.

We gratefully acknowledge the support of the Medical Research Council (Grant G9205639CA) and the Musson Fund of the North Staffordshire Hospital. We also thank Professor R. Wolf and Dr Smith for helpful discussions and advice in establishing CYP2D6 assays, the consultant pathologists of the North Staffordshire Hospital and Dr S. Pemble and Dr J. Taylor for helpful discussions and access to unpublished data.

### References

ANDERSON, M.C. (1991). The natural history of cervical intraepithelial neoplasia. *Curr. Obstet. Gynaecol.*, **1**, 124-129.

BOARD, P.G., COGGAN, M., JOHNSTON, P., ROSS, V., SUZUKI, T. & WEBB, G. (1990). Genetic heterogeneity of the human glutathione S-transferases: a complex of gene families. *Pharmacol. Ther.*, **48**, 357-369.

BURGER, M.P.M., HOLLEMA, H., GOUW, A.S.H., PIETERS, W.J.L.M. & QUINT, W.G.V. (1993). Cigarette smoking and human papillomavirus in patients with reported cervical cytological abnormality. *Br. Med. J.*, **306**, 749-752.

BUSBY-EARLE, R.M.C., STEEL, C.M., WILLIAMS, A.R.W., COHEN, B. & BIRD, C.C. (1994). p53 mutations in cervical carcinogenesis - low frequency and lack of correlation with human papillomavirus status. *Br. J. Cancer*, **69**, 732-737.

CRESPI, C.L., PENMAN, B.W., GELBOIN, H.V. & GONZALEZ, F.J. (1991). A tobacco smoke-derived nitrosamine, 4-(methylnitrosamino)-1-(3-pydridyl)-1-butanone, is activated by multiple human cytochrome P450s including the polymorphic human cytochrome P4502D6. *Carcinogenesis*, **12**, 1197-1201.

CROOK, T., WREDE, D., TIDY, J.A., MASON, W.P., EVANS, D.J. & VOUSDEN, K.H. (1992). Clonal p53 mutation in primary cervical cancer: association with human-papillomavirus-negative tumours. *Lancet*, **339**, 1070-1073.

FRYER, A.A., ZHAO, L., ALLDERSEA, J., PEARSON, W.R. & STRANGE, R.C. (1993). Use of site-directed mutagenesis of allele-specific PCR primers to identify the GSTM1 A, GSTM1 B, GSTM1 A, B and GSTM1 null polymorphisms at the glutathione S-transferase, GSTM1 locus. *Biochem. J.*, **295**, 313-315.

GOUGH, A.C., MILES, J.S., SPURR, N.K., MOSS, J.E., GAEDIGK, A., EICHELBAUM, M. & WOLF, C.R. (1990). Identification of the primary gene defect at the cytochrome $P_{450}$ CYP2D6 locus. *Nature*, **347**, 773-776.

GRAM, I.T., AUSTIN, H. & STALSBERG, H. (1992). Cigarette smoking and the incidence of cervical intraepithelial neoplasia, grade III, and cancer of the cervix uteri. *Am. J. Epidemiol.*, **135**, 341-346.

HEAGERTY, A.H.M., FITZGERALD, D., SMITH, A., BOWERS, B., JONES, P., FRYER, A.A., ZHAO, L., ALLDERSEA, J. & STRANGE, R.C. (1994). Glutathione S-transferase GSTM1 phenotypes and protection against cutaneous malignancy. *Lancet*, **343**, 266-268.

IDLE, J.R., ARMSTRONG, M., BODDY, A.V., BOUSTEAD, C., CHOLERTON, S., COOPER, J., DALY, A.K., ELLIS, J., GREGORY, W., HADIDI, H., HOFER, C., HOLT, J., LEATHART, J., McCRACKEN, N., MONKMAN, S.C., PAINTER, J.E., TABER, H., WALKER, D. & YULE, M. (1992). The pharmacogenetics of chemical carcinogenesis. *Pharmacogenetics*, **2**, 246-258.

ISMAIL, S.M., COLCLOUGH, A.B., DINNEN, J.S., EAKINS, D., EVANS, D.M.D., GRADWELL, E., O'SULLIVAN, J.P., SUMMERELL, J.M. & NEWCOMBE, R.G. (1989). Observer variation in histopathological diagnosis and grading of cervical intraepithelial neoplasia. *Br. Med. J.*, **298**, 707-710.

McINDOE, W.A., McLEAN, M.R., JONES, R.W. & MULLINS, P.R. (1984). The invasive potential of carcinoma in situ of the cervix. *Obstet. Gynecol.*, **64**, 451-458.

NAKACHI, K., IMAI, K., HAYASHI, S. & KAWAJIRI, K. (1993). Polymorphisms of the CYP1A1 and glutathione S-transferase genes associated with susceptibility to lung cancer in relation to cigarette dose in a Japanese population. *Cancer Res.*, **53**, 2994-2999.

PAQUETTE, R.L., LEE, Y.Y., WILCZYNSKI, S.P., KARMAKAR, A., KIZAKI, M. & MILLER, C.W. (1993). Mutations of p53 and human papillomavirus infection in cervical carcinoma. *Cancer*, **72**, 1272-1280.

PEARSON, W.R., VORACHEK, W.R., XU, S., BERGER, R., HART, I., VANNAIS, D. & PATTERSON, D. (1993). Identification of class-mu glutathione transferase genes GSTM1-GSTM5 on chromosome 1p13. *Am. J. Hum. Genet.*, **53**, 220-233.

PEMBLE, S., SCHROEDER, K.R., SPENCER, S.R., MEYER, D.J., HALLIER, E., BOLT, H.M., KETTERER, B. & TAYLOR, J.B. (1994). Human glutathione S-transferase theta (GSTT1): cDNA cloning and the characterisation of a genetic polymorphism. *Biochem. J.* (in press).

SEIDEGARD, J., VORACHEK, W.R., PERO, R.W. & PEARSON, W.R. (1988). Hereditary differences in the expression of the human glutathione S-transferase activity on trans-stilbene oxide are due to a gene deletion. *Proc. Natl Acad. Sci. USA*, **85**, 7293-7297.

SIMONS, A.M., PHILLIPS, D.H. & COLEMAN, D.V. (1993). Damage to DNA in cervical epithelium related to smoking tobacco. *Br. Med. J.*, **306**, 1444-1448.

SMITH, C.A.D., GOUGH, A.C., LEIGH, P.N., SUMMERS, B.A., HARDING, A.E., MARANGANORE, D.M., STURMAN, S.G., SCHAPIRA, A.H.V., WILLIAMS, A.C., SPURR, N.K. & WOLF, C.R. (1992). Debrisoquine hydroxylase gene polymorphism and susceptibility to Parkinson's disease. *Lancet*, **339**, 1375-1377.

STRANGE, R.C. (1993). The glutathione S-transferase GSTM1 locus and cancer susceptibility. In *Structure and Function of Glutathione Transferases*, Tew, K., Mannervik, B., Mantle, T.J., Pickett, C.B. & Hayes, J.D. (eds) pp. 160-171. CRC Press: Boca Raton, FL.

WINKELSTEIN, W. (1990). Smoking and cervical cancer - current status: a review. *Am. J. Epidemiol.*, **131**, 945-960.

WOLF, C.R., SMITH, C.A.D., GOUGH, A.C., MOSS, J.E., VALLIS, K.A., HOWARD, G., CAREY, F.J., MILLS, K., McNEE, W., CARMICHAEL, J. & SPURR, N.K. (1992). Relationship between the debrisoquine polymorphism and cancer susceptibility. *Carcinogenesis*, **13**, 1035-1038.

ZHONG, S., HOWIE, A.F., KETTERER, B., TAYLOR, J.B., HAYES, J.D., BECKETT, G.J., WATHEN, C.G., WOLF, C.R. & SPURR, N.K. (1991). Glutathione S-transferase mu locus: use of genotyping and phenotyping assays to assess association with lung cancer susceptibility. *Carcinogenesis*, **12**, 1533-1537.